# Systematic Review of Interventions to Reduce Operating Time in Lung Cancer Surgery

**DOI:** 10.1177/1179554920987105

**Published:** 2021-02-01

**Authors:** Paulien C Hoefsmit, Robert J Cerfolio, Ralph de Vries, Max Dahele, H Reinier Zandbergen

**Affiliations:** 1 Department of Cardiothoracic Surgery, Amsterdam University Medical Center, Amsterdam, The Netherlands; 2 Department of Cardiothoracic Surgery, New York University Langone Health, New York, USA; 3 Medical Library, Vrije Universiteit, Amsterdam, The Netherlands; 4 Department of Radiation Oncology, Amsterdam University Medical Center, Amsterdam, The Netherlands

**Keywords:** Operating room efficiency, operating room time, lung cancer surgery, improvement, lean

## Abstract

**Introduction:**

Operating rooms are a scarce resource but often used inefficiently. Operating room efficiency emerges as an important part of maximizing surgical capacity and productivity, minimizing delays, and optimizing lung cancer outcomes. The operative time (time between patient entering and leaving the operating room) is discrete and the one that the surgical team can most directly influence. We performed a systematic review to evaluate the literature and identify methods to improve the efficiency of the intraoperative phase of operations for lung cancer.

**Methods:**

A literature search (in PubMed, Embase, Cochrane, and Scopus) was performed from inception up to March 9, 2020, according to the methodology described in the Preferred Reporting Items for Systematic Reviews and Meta-Analysis (PRISMA) statement.

**Results:**

We identified 3 articles relevant to the intraoperative phase of lung cancer operating room efficiency. All 3 were consistent in showing clinically relevant time reductions in the intraoperative phase or procedures relevant to this phase. The authors demonstrated that the application of various improvement methodologies resulted in a substantial reduction in operative time, which was associated with a reduction in complications, and improved staff morale.

**Conclusions:**

Our systematic review found that various improvement methodologies have the potential to significantly reduce operative time for lung cancer surgery. This increases the value of lung cancer surgery. These findings are consistent with the wider literature on improving surgical efficiency.

## Introduction

It is estimated that in 2018 18.1 million people had cancer and 9.6 million died from it, with lung cancer being both the most frequent diagnosed cancer (11.6% of all cases) and the most common cause of death from cancer (18.4% of all deaths).^1^ Non-small-cell lung carcinoma (NSCLC) is the dominant type of lung cancer and accounts for about 85% of all cases.^2^ Surgery is the guideline recommended treatment for early-stage NSCLC in fit patients and may have a role in other stages and clinical situations.^3^ Delays in surgery can compromise outcomes including survival.^4-8^ There is limited surgical operating room capacity and in some countries a lack of access to surgery.^9^ In addition, operating rooms are often not used as efficiently as possible, leading to an effective loss of capacity and productivity.^10^ This has led to targeted improvement efforts both in general and lung cancer surgeries.^11-18^ In the current climate of increased attention to health care value,^19^ operating room efficiency emerges as an important part of maximizing surgical capacity and productivity, minimizing delays, and optimizing lung cancer outcomes. Processes of an operation room can be divided into pre-, intra-, and post-operative. The operative time is defined from the time a patient enters until they exit the operating room. It is an easily measured objective metric and the one that the surgeon and the operating team can most directly influence. We performed a systematic review to evaluate the literature on improving the efficiency (reducing the time) of the intraoperative phase of lung cancer operating room use.

## Materials and Methods

A literature search was performed based on the methodology described in the Preferred Reporting Items for Systematic Reviews and Meta-Analysis (PRISMA) statement.^20^

### Search strategy

To identify relevant publications, we conducted systematic searches in the bibliographic databases PubMed, Embase.com, Cochrane Library (Wiley), and Scopus from inception up to March 9, 2020, in collaboration with a medical information specialist (R.d.V.). The full search strategies for all databases can be found in Appendix A (Supplementary materials). Duplicate articles were excluded. All languages were accepted. The references in the identified articles were searched for relevant publications.

### Selection process

Studies were included if they dealt with any method or innovation directed at improving operating room efficiency for lung cancer surgery, were in English, and available as a full article. Two reviewers (P.C.H. and M.D.) independently screened potentially relevant titles and abstracts for eligibility. If necessary, the full-text article was accessed. Differences were resolved through a consensus procedure.

### Data assessment

The full text of the selected articles was obtained for further review. Two reviewers (P.C.H. and M.D.) independently evaluated the methodological quality of the full-text papers and extracted relevant information.

### Data analysis

We performed a qualitative review and summarized the available publications concerning the intraoperative phase of operating room efficiency for lung cancer surgery.

## Results

### Search results

**Figure 1.**
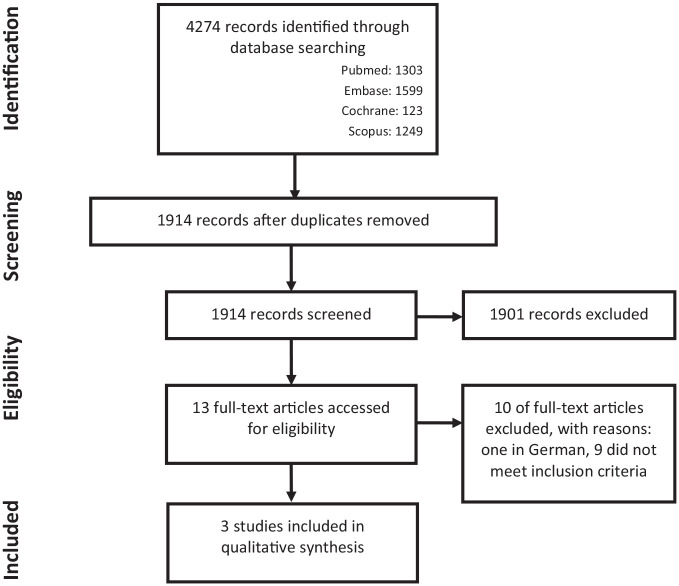
Flowchart of the search and selection procedure of studies.

The results of the search are summarized in Figure 1 and Table 1. The literature search generated a total of 4274 references: 1303 in PubMed, 1599 in Embase.com, 123 in Cochrane, and 1249 in Scopus. However, only 3 articles met all criteria for inclusion in this systematic review.

**Table 1.** Key features and outcomes of included studies.

Author, country	Title	Study type	Number events	Metrics	Improvement method	Main interventions	Outcomes
Cerfolio et al,^21^ USA	Decreasing Time to Place and Teach Double-Lumen Endotracheal Intubation: Engaging Anesthesia in Lean	Interrogation of prospectively maintained single-surgeon database between 1997 and 2017	N = 2940 pulmonary surgeries (n = 2241 lobectomies and n = 566 segmentectomies)	Time to place double-lumen endotracheal tube (DLETT)^a^	Lean and value-stream mapping	Systematic approach to DLETT placement Valued steps of DLETT placement were standardized Development of videos for standardized process	Median time decreased from 13 minutes (first 350 patients) to 45 seconds (last 140 patients; *P* < .0001)
Cerfolio et al,^22^ USA	Decreasing the Pre-incision Time for Pulmonary Lobectomy: The Process of Lean and Value Stream Mapping	Interrogation of retrospective and prospective databases from a single institution between 1997 and 2014 All operations performed by 1 surgeon	N = 2173 pulmonary lobectomies 84% for lung cancer	Pre-incision time^b^ Procedure time^c^ Total operating room time^d^ Time to place double-lumen tube^a^ Patient outcomes (morbidity, mortality)	Lean and value stream mapping Standardizing pre-incision process	In the operating room to skin incision protocols Use of central catheters decreased from 75% to 0%, epidurals from 84% to 3%, arterial catheters from 93% to 4% and Foley catheters reduced from 99% to 11% (*P* < .001 for all) Protocol for DLETT placement Elimination of monitoring devices	Time between operating room entry and incision decreased from a mean of 64 minutes (second cohort of 300 patients) to 37 minutes (last cohort of 373 patients; *P* < .001) Median operating time 155 minutes (first cohort of 300 patients) vs 118 minutes (last cohort of 373 patients) Major morbidity 15.2% vs 5.3% (*P* = .042) and 30-day mortality 3.2% vs 0.26% (*P* = .015)
Iwasaki et al,^23^ Japan	Improvements in Thoracic Surgery Outcomes: A Multi-institutional Collaborative Study	Prospective multi-center study 5 hospitals 2011-2012	N = 745 for all thoracic surgery (before standardization n = 373, after standardization n = 372). Lung lobectomy n = 157 (before standardization n = 74, after standardization n = 83). Partial lung resection n = 388 (before standardization n = 183, after standardization n = 205) Pre- and postoperative time, all cases (before standardization n = 50, after standardization n = 50) Operating time and blood loss, all cases (before standardization n = 373, after standardization n = 372)	Operating room time Pre-operative time^b^ Postoperative time^e^ Blood loss Morale among participants	Procedural standardization and joint meetings between thoracic surgeons and operating room nurses from all institutions	Standardization and protocols for surgical tasks in the preoperative and postoperative period Development of task manual, including videos of surgical techniques	Implementation rate of standardized surgical tasks: 97% Decreased preoperative time after standardization (median of 59-53 minutes, *P* = .008) and postoperative time (median 52-38 minutes, *P* < .0001) Decreased operation times for all thoracic surgeries (146-116 minutes, *P* < .0001), lung lobectomy (228-176 minutes, *P* < .0001), and lung resection (103-92 minutes, *P* = .006) Improved morale among medical staff

Abbreviation: DLETT, double-lumen endotracheal tube.

a Time from start of the anesthesiologist’s laryngoscopy till the taping of it to the face after confirmation.

b Time from entering the operating room to skin incision.

c Time from first incision to closure of last incision.

d Time from patient’s entry to discharge from the operating room.

e Time from closure of last incision to patient exits the operating room.

### Study characteristics

In total, 3 articles were included. Two articles were based on databases from the same surgeon/institution. Multiple end-points were evaluated (Table 1), and several different improvement strategies were used including Lean Methodology, Value Stream Mapping, and Procedural Standardization.

### Outcomes

The key features and outcomes of each study are summarized in Table 1. All 3 articles describe the ability to reduce by a statistically significant and clinically relevant amount, the time taken to perform procedures related to the intraoperative phase of lung cancer surgery, and/or the time taken to perform the surgery itself.

In addition, improvements in major morbidity, 30-day mortality, and staff morale were also reported. Specific components of the process of lean and procedural standardization are summarized in Table 2.

**Table 2.** Specific practical actions resulting from the improvement process.

Author	Specific components
Cerfolio et al^21^	• Prepare equipment prior to intubation • Double-lumen endotracheal tube (DLETT) placement just past vocal cords • Use pediatric bronchoscope to visualize landmarks and correctly position tube • Display bronchoscope view on monitor in the room • Protect patient’s ears and hair with surgical towel; tape tube to face and airway circuit to towel. Securement of DLETT by taping the tube to the patient’s face and the airway circuit to the head wrap • DLETT position not routinely checked again, unless clinical signs of malposition • In selected patients a single-lumen tube was inserted prior to DLETT
Cerfolio et al^22^	• Elimination of axillary rolls, arm boards and beanbags for most patients. Instead, patients positioned on the operating room (OR) table using tape and foam pads • Elimination of routine use of arterial and central catheters • Elimination of routine use of epidural catheters • Elimination of routine use of epidural and Foley catheters • DLETT placement using a systematic approach^21^
Iwasaki et al^23^	• Organization of surgical preparation items and introduction of the cart into the OR • Standardized surgical instrument arrangement in the OR • Signing patients in upon entry to confirm identity • Time-out before surgery (confirmation of patient identity, surgical site, surgical procedure) • Use of compression stockings to prevent deep-vein thrombosis and use of a foot pump until patients start walking • Standardized patient positioning and immobilization • Timing of disinfection and prevention of disinfectant-induced chemical burns • Management of body temperature with a warm-air temperature management unit • Standardization surgical techniques • Ensuring presence of particular surgical instruments • Organization of surgical devices and consistency of nomenclature • Installation of a low-pressure suction device routinely • Standardization postoperative thoracic radiography and its timing

## Discussion

Operating room inefficiency is common. It is estimated to cost the United Kingdom National Health Service (NHS) nearly 300 000 operations a year.^10^ This systematic review identified 3 articles relevant to the intraoperative phase of lung cancer operating room efficiency. Two were from the same senior author (R.J.C.) in the United States and one was a multicenter study from Japan. Overall, the authors demonstrated that the application of various improvement methodologies may result in significant and clinically relevant reductions in the time that an operating room is occupied for a patient’s surgery, a reduction in complications, and an improvement in staff morale. Although none of the papers reported a randomized controlled study, which limits the strength of the evidence, all 3 papers were consistent in showing clinically relevant time gains and included substantial numbers (hundreds to >2000) of patients and procedures. The general process improvement methodologies used in the papers combined with specific improvement actions can be readily adopted by interested parties and used to stimulate improvements in their own service. The magnitude of the time gains reported by some of the studies in this review is substantial. In some cases, it could mean that there is enough time to perform at least one whole additional surgery on the list.

Organizational change has typically been associated with high failure rates.^24^ In addition to the methodology itself, factors like a high level of motivation, buy-in, and competent execution are necessary to achieve improvement. Previous work demonstrates that the following factors can influence the success of clinical improvement programs: awareness, alignment, and engagement among all involved surgical team members, standardization, leadership, and guidance.^12^ Barriers for improvement programs include dogma and resistance to eliminating preferred clinical steps despite evidence showing they add no value.^22^ Implementing change in organizations takes time and persistence.

Improving efficiency in the operating room leads to better financial performance due to enhanced revenue and reduced costs.^12,13,15,25^ Second to room and board costs, operating rooms are the most expensive component of surgical care.^26^ Although the articles in this systematic review did not report data concerning the effect of improving efficiency in the operating room on financial performance, Cerfolio et al^13^ reported that the costs of implementing a multidisciplinary improvement program to improve operating room efficiency were US $1298 per day and an estimated return on investment of US $19 500 per day.

Outside of lung cancer surgery, and the intraoperative phase, there is a wider body of literature about increasing operating room efficiency. This includes other improvement methods like Six Sigma, Total Quality Management, Plan-Do-Study-Act, Plan-Do-Check-Act, Statistical Process Control, and Statistical Quality Control.^17^ These have been shown to be effective in both general and cancer surgeries.^14-18,27^ Van den Heuvel et al^27^ reported that using Six Sigma to improve the operating room admissions process alone and reducing the start time by an average of 9 minutes, allowed an additional 400 surgeries per year and a net saving of >US $273 000 in a 384-bed community hospital.

There are a number of limitations in this systematic review. We could only identify a very small number of papers. This was not completely unexpected given the small number of surgical process improvement papers (n = 23 and n = 34) found to be suitable for inclusion in reviews with a much wider focus on all steps in surgical care and all types of surgery.^16,17^ In addition, there may be bias if unsuccessful efforts to increase operating room efficiency have not been published. In choosing to limit the review to the intraoperative phase of the surgical process, the scope of our conclusions may also be limited. There are many other steps that also require attention if the goal is to maximize the overall efficiency and value of surgery. For example, delays in starting the first operation of the day can lead to inefficiencies such as cancelations and delays at the end of the day.^28,29^ Addressing this can result in improved workflows and a reduction in wasted operating room time.^29^ In another study, reducing operating room turnover time potentially freed up an extra 70 minutes per day, which could be used for performing another surgery without the costs of overtime staff.^13^ We also note that the articles included in the review do not specifically address the influence of learning curve, surgical method, or teaching on operating room efficiency. David et al^30^ have reported that greater experience with video-assisted thoracoscopic surgery (VATS) affects performance and can lead to a reduction in costs and resource utilization. Increased experience with VATS results in a lower conversion rate to open thoracotomy.^31^

## Conclusions

In conclusion, we identified a small number of articles that clearly demonstrated the potential to improve the operative time of patients undergoing lung cancer surgery. This increases operating room efficiency and in so doing, the value of lung cancer surgery. This is consistent with the wider literature on improving surgical efficiency.

## Supplemental Material

sj-pdf-1-onc-10.1177_1179554920987105 – Supplemental material for Systematic Review of Interventions to Reduce Operating Time in Lung Cancer Surgery Click here for additional data file. Supplemental material, sj-pdf-1-onc-10.1177_1179554920987105 for Systematic Review of Interventions to Reduce Operating Time in Lung Cancer Surgery by Paulien C Hoefsmit, Robert J Cerfolio, Ralph de Vries, Max Dahele and H Reinier Zandbergen in Clinical Medicine Insights: Oncology
